# Investigating association between inflammatory bowel disease and rotavirus vaccination in a paediatric cohort in the UK

**DOI:** 10.1017/S0950268823000936

**Published:** 2023-06-09

**Authors:** Aidan Flatt, Thomas Inns, Kate M. Fleming, Miren Iturriza-Gómara, Daniel Hungerford

**Affiliations:** 1 Institute of Population Health, Department of Public Health, Policy and Systems, University of Liverpool, Liverpool, UK; 2 NIHR HPRU in Gastrointestinal Infections at University of Liverpool, Liverpool, UK; 3 Institute of Infection, Veterinary and Ecological Sciences, Department of Clinical Infection, Microbiology and Immunology, University of Liverpool, Liverpool, UK; 4 Centre for Vaccine Innovation and Access, PATH, Geneva, Switzerland

**Keywords:** Crohn’s disease, infectious disease, inflammatory bowel disease, paediatric, rotavirus vaccine, survival analysis, ulcerative colitis, vaccine safety

## Abstract

In the UK, the incidence and prevalence of inflammatory bowel disease (IBD) is increasing in paediatric populations. Environmental factors including acute gastroenteritis episodes (AGE) may impact IBD development. Infant rotavirus vaccination has been shown to significantly reduce AGE. This study aims to explore the association between vaccination with live oral rotavirus vaccines and IBD development. A population-based cohort study was used, analysing primary care data from the Clinical Practice Research Datalink Aurum. Participants included children born in the UK from 2010 to 2015, followed from a minimum of 6 months old to a maximum of 7 years old. The primary outcome was IBD, and the primary exposure was rotavirus vaccination. Cox regression analysis with random intercepts for general practices was undertaken, with adjustment for potential confounding factors. In a cohort of 907,477 children, IBD was recorded for 96 participants with an incidence rate of 2.1 per 100,000 person-years at risk. The univariable analysis hazard ratio (HR) for rotavirus vaccination was 1.45 (95% confidence interval (CI) 0.93–2.28). Adjustment in the multivariable model attenuated the HR to 1.19 (95% CI 0.53–2.69). This study shows no statistically significant association between rotavirus vaccination and development of IBD. However, it provides further evidence for the safety of live rotavirus vaccination.

## Introduction

Inflammatory bowel disease (IBD) encompasses conditions of the gastrointestinal tract which cause chronic and relapsing inflammation [1] and presents significant physical and mental health morbidities to diagnosed patients, alongside considerable costs to healthcare [2, 3].

IBD prevalence in the UK is increasing, with estimates of 142 cases of IBD per 10,000 population, an increase of over 30% since 2006 [4]. Paediatric incidence has seen a similar increase from 7.71 per 100,000 per year to 10.54 between 2013 and 2017 [5]. In children, peak onset of IBD is in adolescence; however, around 20% of under 18s with IBD will have been diagnosed before their 10th birthday [6].

Environmental factors including infections, antibiotics, and vaccinations have been suggested as possible causes [7–9]. Immune dysregulation causing gut inflammation appears to play a significant role in the development of chronic IBD [10]. The impacts of bacterial sources of gastrointestinal infection and the effects these have on the enteric bacterial microbiome in IBD are well documented [11, 12]. However, the status of the enteric virome in IBD appears less well understood [13].

Rotavirus causes acute gastroenteritis episodes (AGE) characterised by vomiting, diarrhoea, fever, and tiredness [14]. Rotavirus is the most common cause of gastroenteritis in children under 2 years old, and remains the most common viral cause of gastroenteritis in paediatric cases globally in unvaccinated groups [15].

Rotavirus vaccination with the live-attenuated rotavirus vaccine (Rotarix, GlaxoSmithKline) given in two doses at 8 and 12 weeks of age was introduced into the routine immunisation schedule in the UK in 2013. Infant rotavirus vaccination has resulted in a significant reduction in severe disease and hospitalisations due to rotavirus infection and a reduction in infection rates [16]. In England, rotavirus vaccine effectiveness is high at 85% in children aged under 12 months [17].

Given a substantial improvement in rates of rotavirus infection following vaccination, the possible role of vaccination in the development of paediatric IBD can be explored. Furthermore, it has been posited that the rotavirus vaccination itself, being a live oral vaccine, has biological plausibility in mediating gut inflammation [8]. Other live vaccinations have been shown to increase the risk of developing IBD, including poliomyelitis vaccination and BCG vaccination [18].

Current literature shows varied results where different study designs and a range of settings make for challenging comparisons. Episodes of acute gastroenteritis have been shown to increase the odds of incident IBD in populations in the UK, USA, and Sweden [19–22], and decrease the risk in populations in Spain [7]. These studies largely use adult populations and retrospective study designs. A Malaysian study on children under 18 years old reported an odds ratio of 6.93 for developing IBD following AGE, and a protective effect of the rotavirus vaccination against IBD (odds ratio 0.14) [23]. In a nested case–control study of children under 10 years old in North America, Liles et al. reported no statistically significant effect of rotavirus vaccination exposure on the odds of developing IBD [8].

The conflicting evidence surrounding the relationship between rotavirus infection and vaccination, and the development of IBD in paediatric populations highlights the need for further research in this area. This study aims to explore the association between paediatric IBD and rotavirus vaccination in a birth cohort of children followed for up to 7 years in the UK.

## Methods

### Study design and setting

A population-based cohort study was undertaken using data from the Clinical Practice Research Datalink (CPRD) Aurum. Primary care level data is included in the set from over 11 million patients registered at almost 700 general practice (GP) surgeries across the UK, and includes health data on demographics, investigations, diagnoses, prescriptions, contact with healthcare professionals, and referrals to secondary care [24].

### Participants

Anonymised data were extracted from the CPRD dataset, where follow-up began at 6 months of age (the oldest eligibility to receive the rotavirus vaccination), ensuring that participants did not move between groups of differing vaccination status.

Follow-up ceased for children recorded to have incident IBD, or those who moved out of the GP practice, died, or reached their seventh birthday. Data extraction took place in 2020. As such, follow-up to 7 years was undertaken as this was the maximum time a child born in the rotavirus vaccine era (2013 onwards) could be followed up for. This allowed for the equalisation of the follow-up time between the comparator groups.

Children born prior to the rotavirus introduction in the UK were included to ensure power and balance the comparator groups. This was due to vaccine uptake rapidly increasing following routine introduction to nearly 90% in early 2014 [25]. Therefore, to compare low numbers of unvaccinated children (10%) with high numbers of vaccinated children (90%), plus considering the effects of potential differences in healthcare-seeking behaviours, would risk possible confounder-dependent associations between the outcome and chance of being vaccinated [26]. As such, 2010 was chosen as the earliest entry point to minimise the temporal bias created by time dislocation between groups, with a maximum entry point from children born in 2015.

As a result, health records for 926,013 children born between 2010 and 2015 were extracted, with the total reduced to 907,477 following censoring at 7 years old.

### Study variables

The outcome measure was first IBD diagnosis, discerned from the relevant medical codes in the dataset [4, 27] (Supplementary Table S1). The exposure variable was measured as a binary variable of administration of one or more doses of the Rotarix vaccination against no doses given, as the majority of UK children receive both doses (88.3% vs. 6% receiving only one dose) [25] and the immunity conferred from one dose is comparable to two [28].

Potentially confounding variables were identified a priori. Adjustments for sex and age were made. Adjustment for the year of birth accounted for changes in IBD diagnosis rates over time. A measure of deprivation was made using the Index of Multiple Deprivation (IMD) scores. Healthcare-seeking behaviours and vaccine hesitancy were identified as potential confounders. To account for these, the average rate of GP consultations per participant was adjusted for, and a sensitivity analysis was conducted to restrict the cohort to only include individuals who had received the Diptheria-Tetanus-Polio (DTP) vaccination. The DTP vaccine has been a long-standing component of the UK immunisation schedule, so can be used as a proxy measure of vaccine hesitancy and healthcare-seeking behaviour [26].

### Sample size

Using the exponential test comparing two independent hazard rates in Stata V14, the study power was estimated for the primary objective using group sizes of 537,516 (vaccinated) and 343,113 (unvaccinated) [29]. The prevalence of IBD in children aged 6–10 years is estimated to be 0.025% [5]. A follow-up of up to 10 years was specified, with a loss to follow-up of 0.4 over 5 years in the vaccinated group and 0.2 in the unvaccinated group.

Using two-sided power calculations and an alpha of 0.05, the population size power was estimated at a range of hazard ratios (HRs) and IBD prevalence rates. For example, power was estimated at 0.85 for a 25% reduction in hazard.

### Statistical methods

Descriptive analyses were undertaken for the exposure and outcome variables, sex, year of birth, region of residence, IMD score, DTP vaccine status, and GP consultations per year. The distribution of these between the unexposed and exposed groups was undertaken using hypothesis testing of the null hypothesis, where Chi-squared testing was used for categorical variables, *t*-tests for normally distributed continuous variables, and Wilcoxon Rank-Sum testing for non-normally distributed continuous variables. *p*-values were calculated with a significance level of 5%.

Cox regression survival analysis was undertaken to explore the relationship between the exposure and the outcome. Kaplan–Meier survival plots were generated for the primary exposure variable of rotavirus vaccination status. The proportional hazards assumption was tested by inspecting the Kaplan–Meier curves generated, creating a plot against log time, and testing the Schoenfeld residuals. The log-rank hypothesis test was used to test the null hypothesis of there being no difference in the probability of incident IBD in the vaccinated and unvaccinated groups at any point in time.

Random intercepts for GP practices were included in the survival analyses to account for unmeasured potential differences in outcome measurement between GP practice clusters.

Univariable analysis for the exposure variable was undertaken. Multivariable survival analysis was undertaken with the variables identified a priori, with the exception of region as the model lacked the power to assess this due to the rarity of the outcome. HRs with 95% confidence intervals (CIs) were generated from these analyses.

Two sensitivity analyses were undertaken. The first restricted the cohort to those who had been given the DTP vaccination to account for potential differences in healthcare-seeking behaviours and vaccine hesitancy. The second sensitivity analysis did not censor the cohort at 7 years old to investigate possible effects on older children aged 7–10 years old. The same multivariable survival analyses were undertaken to assess the effects of these sensitivity analyses.

## Results

### Participants

Following censoring at 7 years of age, the total number of participants was 907,477. In the overall cohort, there were 464,735 males (51.2%) and the highest proportion of participants were born in 2010 (18.7%). Regionally, the highest proportion of participants lived in the South West (20.7%). 23.3% of the total participants had an IMD score of 5 (most deprived). A full set of cohort characteristics and distribution of variables can be seen in Table 1.

The distribution of sex by vaccination status was not statistically significant (*p* > 0.05). The explanatory variable of year of birth was significantly associated with the exposure variable (*p* < 0.001) explained by the introduction of the rotavirus vaccination into the UK routine schedule in 2013. Region was also statistically significantly associated with rotavirus vaccination (*p* < 0.001). The mean rate of GP consultations per year was significantly higher per participant in the vaccinated group compared to the unvaccinated group (*p* < 0.001).

The total number of IBD diagnoses in children followed up from 6 months to a maximum of 7 years of age was 96. There were 4,577,436 person-years-at-risk for the overall number of participants in this cohort. The incidence rate of IBD was 2.1 per 100,000 person-years-at-risk. Amongst those with an IBD diagnosis, 63.5% were male and 36.5% were female.

In the exposed group, there were 32 cases of IBD diagnosis, and in the unexposed group, there were 64 cases. IBD diagnoses were not significantly associated with rotavirus vaccine status (*p* = 0.479). The average age of diagnosis of IBD was 4.5 years old, with no statistically significant difference between vaccinated and unvaccinated groups (*p* = 0.051).

### Survival analysis

**Figure 1.**
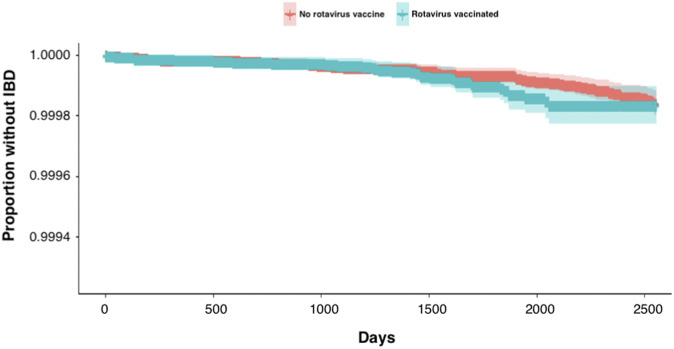
Kaplan–Meier survival plot of IBD against rotavirus vaccination status (shaded ribbons represent 95% confidence intervals).

A Kaplan–Meier curve was generated to visualise the association between IBD incidence survival and rotavirus vaccination over time, seen in Figure 1. The hazards are proportional in the vaccinated and unvaccinated groups, with the 95% CIs overlapping throughout.

**Table 1.** Baseline descriptive statistics of the population cohort

Variable		Overall (*n* = 907477)		Rotavirus unvaccinated (*n* = 568524)		Rotavirus vaccinated (*n* = 338953)		*p*-value
		*n*	%	*n*	%	*n*	%	
Sex	M	464735	51.2	291258	51.2	173477	51.2	0.644
	F	442738	48.8	277263	48.8	165475	48.8	
Year of birth	2010	169436	18.7	167965	29.5	1471	0.4	<0.001
	2011	164038	18.1	162170	28.5	1868	0.6	
	2012	159315	17.6	156861	27.6	2454	0.7	
	2013	146007	16.1	55397	9.7	90610	26.7	
	2014	138101	15.2	14425	2.5	123676	36.5	
	2015	130580	14.4	11706	2.1	11887	35.1	
Region of residence	North East	34629	3.8	21457	3.8	13172	3.9	<0.001
	North West	135578	14.9	8511	15.0	50461	14.9	
	Yorkshire	32312	3.6	20405	3.6	11907	3.5	
	East Midlands	22327	2.5	14077	2.5	8250	2.4	
	West Midlands	151393	16.7	94659	16.7	56734	16.7	
	East of England	52012	5.7	31979	5.6	20033	5.9	
	London	113788	12.5	71444	12.6	42344	12.5	
	South East	111466	12.3	69187	12.2	42279	12.5	
	South West	187842	20.7	119247	21.0	68595	20.2	
	Wales	65738	7.2	40668	7.2	25070	7.4	
IMD score	1	177369	19.6	109172	19.2	68197	20.1	<0.001
	2	162821	18.0	99965	17.6	62856	18.6	
	3	166749	18.4	103917	18.3	62832	18.6	
	4	188394	20.8	119125	21.0	69269	20.5	
	5	211315	23.3	135830	23.9	75485	22.3	
DTP vaccine status	Vaccinated	869830	95.9	531339	93.5	338491	99.9	<0.001
	Not vaccinated	37647	4.1	37185	6.5	462	0.1	
IBD record	No	907381	100	568460	100	338921	100	0.479
	Yes	96	0	64	0	32	0	

	Mean	SD	Mean	SD	Mean	SD	
Age of IBD diagnosis in years	4.45	2.06	4.74	2.18	3.87	1.70	0.051
Days of follow-up	1,842.37	773.38	2000.50	810.08	1577.14	623.17	<0.001
GP consultations per year	6.9	4.67	6.55	4.71	7.48	4.55	<0.001

**Table 2.** Univariable and multivariable analyses using mixed-effects Cox regression with random GP intercepts

		Univariable		Multivariable		*p*-value
		HR	95% CI	aHR	95% CI	
Rotavirus vaccination	Unvaccinated	*ref*	*ref*	*ref*	*ref*	*ref*
	Vaccinated	1.45	0.93–2.28	1.19	0.53–2.69	0.675
Sex	Male	*ref*	*ref*	*ref*	*ref*	*ref*
	Female	0.6	0.4–0.91	0.68	0.45–1.03	0.066
Year of birth	2010	*ref*	*ref*	*ref*	*ref*	*ref*
	2011	1.57	0.8–3.08	1.82	0.92–3.6	0.086
	2012	1.74	0.89–3.4	2.38	1.19–4.73	0.014
	2013	1.77	0.86–3.67	2.45	0.99–6.05	0.053
	2014	2.13	0.98–4.66	3.09	1.03–9.23	0.044
	2015	2.47	1.05–5.79	3.97	1.25–12.63	0.019
IMD score	1	*ref*	*ref*	*ref*	*ref*	*ref*
	2	0.76	0.36–1.57	0.72	0.35–1.5	0.383
	3	1.27	0.67–2.4	1.22	0.64–2.32	0.541
	4	1.04	0.54–2.01	0.99	0.51–1.91	0.974
	5	1.44	0.79–2.62	1.31	0.72–2.39	0.373
Number of consultations		1.02	1.02–1.02	1.02	1.02–1.03	<0.001

aHR, adjusted hazard ratio; CI, confidence interval; HR, hazard ratio.

**Table 3.** Sensitivity analyses with random GP intercepts, undertaken for cohort restricted to those with DTP vaccination, and IBD population not censored to under 7 years old

Sensitivity analysis	Univariable		Multivariable		*p*-value
	HR	95% CI	aHR	95% CI	
Original survival analysis	1.45	0.93–2.28	1.19	0.53–2.69	0.675
Censored to only those with DTP vaccination	1.44	0.91–2.27	1.10	0.48–2.51	0.827
IBD population not censored to under seven years old	1.48	0.95–2.31	1.32	0.59–2.96	0.495

aHR, adjusted hazard ratio; CI, confidence interval; HR, hazard ratio.

Undertaking mixed-effects Cox regression with random intercepts for GP practices generated the results shown in Table 2. In the univariable analysis, the HR for the association between IBD and rotavirus vaccination was 1.45 (95% CI 0.93–2.28). In the multivariable model including the variables identified a priori the HR reduced to 1.19 (95% CI 0.53–2.69).

In participants born in 2014 and 2015, the adjusted HRs of 3.09 and 3.97 respectively were statistically significant at the 5% significance level (*p* < 0.05). The number of GP consultations as a marker of healthcare-seeking behaviour also showed a statistically significantly higher hazard of incident IBD in both models.

Sex, year of birth prior to 2014, and IMD score did not statistically significantly change the hazard of incident IBD in this cohort in both univariable and multivariable models.

### Sensitivity analyses

The results from the sensitivity analyses are shown in Table 3. Restricting the cohort to only those participants who had been given the DTP vaccination reduced the HR of rotavirus vaccination to 1.10 (95% CI 0.48–2.51). Undertaking a sensitivity analysis on the population not censored to exit the cohort on their 7th birthday generated an HR of 1.32 (95% CI 0.59–2.96).

## Discussion

This study found no significant association between rotavirus vaccination and incident paediatric IBD in a UK cohort.

The results from this study are in keeping with those from Liles et al. which provides recent findings from a comparable paediatric population in the USA [8]. These results add consistency to the evidence that the rotavirus vaccination appears safe to use in the UK paediatric population and does not support IBD being an unintended consequence of vaccination. However, direct comparison with Liles et al. is challenging given the differing study designs and outcome measures.

This study provides contrasting results to those of Lee et al., whose findings reported an 86% protective effect of the rotavirus vaccine against the development of IBD in Malaysian children aged under 18 [23]. These findings are likely to differ given disparities between the study populations where there is likely differing prevalence of infectious diseases and differences in the routine administration of the rotavirus vaccination, as in Malaysia it is not given in the routine schedule [30].

Comparisons to other literature in this subject area are challenging, where existing publications use adult populations, AGE as the exposure variable, and significantly different study designs and methodologies [19–22].

Findings from this study contribute to the evidence surrounding the safety of the rotavirus vaccination in UK children, where no significant effects on the development of IBD have been elicited in association with the administration of the vaccination during the first 6 months of age.

### Study strengths

This study used a large dataset with broad coverage of children in the UK, increasing the generalisability of the results as the cohort is highly representative of the UK paediatric population. The representativeness is further increased by using this UK-based setting where healthcare is free at the point of use. Additionally, the reliability of the data source is a strength of this study, whereby regular checks of quality and validation of the data are undertaken by CPRD. Furthermore, rotavirus and DTP vaccine uptake figures within this cohort are similar to that reported in the UK national COVER data, providing further confidence in the data quality [31].

The study design is also a significant strength, as the majority of comparable research uses case–control studies subject to recall bias. By using a prospective population-based cohort design for this study, this bias was minimised.

The statistical analysis methods further strengthen this study. The use of sensitivity analyses provided confidence that the initial cohort analysis was robust, and that reducing the population to only those who had received the DTP vaccination to account for potential differences in healthcare-seeking behaviours and as a proxy for vaccine hesitancy provided similar results, allowing us to report on the higher powered main cohort.

Potential confounders were included in the multivariable analysis and were identified a priori, ensuring that no significant variables were excluded in the lead-up to the analysis, which is a potential risk in stepwise methods of selection of variables [32]. Year of birth was included as a variable and allowed adjustment for the potential effects of changing diagnostic rates over time. Significant differences in healthcare-seeking behaviours between the exposed and unexposed groups in this study were observed. The number of GP consultations per year was adjusted for in the multivariable models in this study. Adjustment for healthcare-seeking behaviours is lacking in much of the currently published literature and highlights a relative strength of this study.

### Study limitations

This study is limited by its use of a population preceding the peak diagnosis age of IBD in the UK. This is on account of the timing of the introduction of the rotavirus vaccination into the UK routine vaccination schedule. Ceasing follow-up on the participants’ seventh birthday likely resulted in a lower number of outcomes. This also meant that a smaller number of variables could be used in the analysis, and that variables such as region needed exclusion. Instead, a measure of deprivation was prioritised and random intercepts for GP practices were included, between which there are likely to be larger differences in health status and access to services compared to between regions.

Lengthening the follow-up time to include older children may increase the number of outcomes; however, this would include the period covering the Covid-19 pandemic. Atypical healthcare access, healthcare-seeking behaviours, diagnoses of conditions, and infectious disease transmission during this time may significantly affect any subsequent data collected during this period. The current follow-up time may have resulted in a lower number of outcomes, but represents a stable population from which data was collected during a steady period in time.

Given IBD is a chronic disease, it is possible that children with early IBD did not yet have a diagnosis. Further research in this area could include codes relating to investigation orders, including biomarkers of gut inflammation, such as faecal calprotectin, to capture potential cases not yet diagnosed.

Antibiotic use in the treatment of acute bacterial gastroenteritis may confound the results in existing studies assessing exposure to all-cause gastroenteritis when unadjusted for antibiotic use. Antibiotic prescription for gastroenteritis is less common in this primary care research population, given viral causes will make up the majority of cases. Further attention on this subject should consider that rotavirus vaccination is shown to reduce antibiotic prescribing for AGE in primary care [33]. As such vaccination may influence IBD incidence through reductions in both infection and antibiotic prescribing.

## Conclusion

Within this study of a paediatric cohort in the UK, no statistically significant association between rotavirus vaccination and the development of IBD was found following mixed-effects Cox regression analysis. The findings from this study do not advocate for a change in the current UK public health policy surrounding rotavirus vaccination, but contribute evidence to demonstrate the safety of this live, orally administered vaccination.

These findings are in keeping with a recent similar study using a comparable US population assessing the same exposure and outcome variables [8]. However, comparison to the existing literature is challenging due to a variety of study designs, the majority of which are subject to significant recall bias.

Future research would benefit from a repeat analysis of the dataset with a longer follow-up period into the teenage years, and the use of cohort study designs in non-UK populations to allow more comparisons to be made between study settings.

## Data Availability

The datasets used in this study were extracted from Clinical Practice Research Datalink (CPRD) following CPRD approval of the study protocol (20_024: available at https://cprd.com/protocol/) and through a multi-study license and data sharing agreement between the University of Liverpool and CPRD. The authors are not authorised to share the datasets and are obliged to destroy the datasets according to the data-sharing agreement between the University of Liverpool and CPRD.

## Supplementary material

For supplementary material accompanying this paper visit http://doi.org/10.1017/S0950268823000936. click here to view supplementary material
