# Supplementary material for: Investigating association between inflammatory bowel disease and rotavirus vaccination in a paediatric cohort in the UK
Source: Epidemiol Infect. 2023 Jun 9;151:e103. doi: 10.1017/S0950268823000936 (PMC10311680; doi:10.1017/S0950268823000936)
Supplement: Supplementary file 1 [file S0950268823000936sup001.docx]

**Supplementary Material**

Journal: Epidemiology and Infection

Title: Investigating association between inflammatory bowel disease and rotavirus vaccination in a paediatric cohort in the UK

Authors: Aidan Flatt^1,2^, Thomas Inns^2^, Kate M Fleming^1^, Miren Iturriza-Gomara^2,3,4^, Daniel Hungerford^2,3*^

Affiliations

1. Institute of Population Health, Department of Public Health, Policy & Systems, University of Liverpool, Liverpool, UK

2. NIHR HPRU in Gastrointestinal Infections at University of Liverpool, Liverpool, UK

3. Institute of Infection, Veterinary & Ecological Sciences, Department of Clinical Infection, Microbiology and Immunology, University of Liverpool, Liverpool, UK

4. Centre for Vaccine Innovation and Access, PATH, Geneva, Switzerland

Corresponding author: Daniel Hungerford, Institute of Infection, Veterinary & Ecological Sciences, Department of Clinical Infection, Microbiology and Immunology, University of Liverpool, The Ronald Ross Building, 8 West Derby Street, Liverpool, L69 7BE

E: d.hungerford@liverpool.ac.uk | T: 0151 795 1455

**Table S1; CPRD Aurum medical codes and product codes used for exposure and outcome variable ascertainment**

| **Medical codes for rotavirus vaccine** | "2535217016", "2245021000000115", "2244981000000119", "2245061000000111” |
| --- | --- |
| **Product codes for rotavirus vaccine** | "3922441000033114", "5989741000033114", "12349441000033117" |
| **Inflammatory bowel disease medical codes** | "302322010", "497569010", "302937016", "601091000006115", "302939018", "302940016", "2532958014", "179571000006119", "302941017", "179521000006115", "488238014", "2532950019", "1495442018", "396357012", "601031000006119", "302946010", "179501000006113", "56765016", "56770011", "302947018", "1222351011", "303761010", "309744019", "309833017", "411543016",  "302953018", "107644019", "496332018", "496249010", "2532953017",  "435370011", "302956014", "85891000006115", "85901000006116",  "2872721013", "2579429013", "696071000006114", "85931000006112",  "302959019", "302961011", "302962016", "303762015", "309743013",  "309836013", "2269901000000115", "2891431000006118", "41137017",  "886291000006112", "906051000006118", "3047391000006119", "3414711000006118","3420891000006119","4784111000006112","  3047411000006119", "2559801000006116", "4785581000006112", "3113551000006113", "3316751000006117", "3316811000006110",  "2559781000006115", "3316801000006112", "3113541000006111", "4784091000006115", "3414701000006116", "6853131000006115", "3047421000006110", "4809351000006111", "2621151000006116", "3420881000006117", "3414681000006119","6853111000006114",  "2621161000006119", "2559821000006114", "4808981000006112", "906191000006113", "3553391000006113", "3346691000006114", "3346681000006111", "3351341000006118" |
